# Transmission Shifts Underlie Variability in Population Responses to *Yersinia pestis* Infection

**DOI:** 10.1371/journal.pone.0022498

**Published:** 2011-07-25

**Authors:** Michael G. Buhnerkempe, Rebecca J. Eisen, Brandon Goodell, Kenneth L. Gage, Michael F. Antolin, Colleen T. Webb

**Affiliations:** 1 Department of Biology, Colorado State University, Fort Collins, Colorado, United States of America; 2 Division of Vector-Borne Infectious Diseases, Centers for Disease Control, Fort Collins, Colorado, United States of America; University of Hyderabad, India

## Abstract

Host populations for the plague bacterium, *Yersinia pestis*, are highly variable in their response to plague ranging from near deterministic extinction (i.e., epizootic dynamics) to a low probability of extinction despite persistent infection (i.e., enzootic dynamics). Much of the work to understand this variability has focused on specific host characteristics, such as population size and resistance, and their role in determining plague dynamics. Here, however, we advance the idea that the relative importance of alternative transmission routes may vary causing shifts from epizootic to enzootic dynamics. We present a model that incorporates host and flea ecology with multiple transmission hypotheses to study how transmission shifts determine population responses to plague. Our results suggest enzootic persistence relies on infection of an off-host flea reservoir and epizootics rely on transiently maintained flea infection loads through repeated infectious feeds by fleas. In either case, early-phase transmission by fleas (i.e., transmission immediately following an infected blood meal) has been observed in laboratory studies, and we show that it is capable of driving plague dynamics at the population level. Sensitivity analysis of model parameters revealed that host characteristics (e.g., population size and resistance) vary in importance depending on transmission dynamics, suggesting that host ecology may scale differently through different transmission routes enabling prediction of population responses in a more robust way than using either host characteristics or transmission shifts alone.

## Introduction

Plague, caused by the bacterium *Yersinia pestis*, remains a public health concern because of its high virulence in multiple mammal species, including humans, and its role in past pandemics in humans. Despite its historical importance and the continued threat of human cases, plague is primarily a disease of rodents and their fleas. Consequently, humans are at greatest risk of exposure to *Y. pestis* during plague epizootics when rodent hosts die in large numbers increasing potential exposures to sick or dead animals and infectious fleas [1]. Thus, understanding outbreaks in rodents may aid in prediction, control and prevention of human cases.

However, rodent species show high variability in their population-level response to plague infection, and the mechanisms that determine outbreak conditions are not fully understood. The variability in host response can be compartmentalized into two classes: either enzootic (i.e., low probability of extinction despite persistent infection in a population) or epizootic (i.e., high probability of extinction due to plague). This classification enables predictions that can be based on observable intra-population dynamics rather than invoking landscape-level maintenance mechanisms involving the interaction of plague dynamics in multiple species [2]–[4].

Previous research on plague dynamics depended on observation of host characteristics to differentiate between epizootic and enzootic populations. For example, enzootic hosts, such as great gerbils (*Rhombomys opimus*) in Kazakhstan, show high levels of prolonged resistance (40–60% of hosts; [4]) while epizootic hosts, like black-tailed prairie dogs (*Cynomys ludovicianus*), rarely survive plague infection [5]. In gerbils, disease prevalence also exhibits a threshold behavior with host abundance where plague fails to persist below the threshold [6], [7] and prevalence increases with host abundance above the threshold [8]. While these observations aid in prediction, they largely ignore one of the key components in plague dynamics: fleas and their effect on transmission.

Here, we propose that shifts from enzootic to epizootic dynamics could be accounted for by variation in the relative strength of alternative transmission routes, an avenue of plague research that has received relatively little attention. Theoretical work supports the notion that heterogeneities in transmission rates determine population disease dynamics [9]–[11]. For almost a century, a single transmission route depending upon blocked fleas (i.e., formation of a biofilm in a flea's midgut resulting in continued feeding attempts and subsequent regurgitation of bacteria) has been the dominant transmission paradigm for plague [4], [12]. This focus has left other transmission routes relatively unexplored, but a recent modeling study questioned the role of blocked-fleas in plague dynamics sparking interest in alternative transmission routes [13].

Experimentally studying transmission routes in natural systems is nearly impossible, but laboratory experiments have identified effective transmission routes that could also affect population responses to plague infection. In particular, early-phase transmission by un-blocked fleas (i.e., transmission immediately following an infectious blood meal) has been shown to be a viable alternative to blocked-flea transmission in several flea species under laboratory conditions [14]–[19]. A “booster” feed infection cycle (i.e., continued blood meals on infectious hosts that boost the density of *Y. pestis* in the flea) allows for the maintenance of infection levels in fleas and increases infectious duration for early-phase transmission [20]. In addition, the role of transmission from external reservoirs, such as infected, questing (i.e., host-seeking) fleas [4], [21], [22] or infected carcasses [23], is largely unstudied but potentially important. Indeed, infected questing fleas have survived for over a year in the field [24]–[27], and viable *Y. pestis* has survived in carcasses and soil for several days under both field and laboratory conditions [28]–[30].

In order to simultaneously consider how multiple transmission routes interact to determine plague dynamics, we present a general model of *Y. pestis* dynamics that incorporates three routes of plague transmission: 1) the booster-feed infection cycle; 2) the build-up of infectious, questing fleas; and 3) contact with carcass-derived material. We parameterize the model for an epizootic host, the black-tailed prairie dog, and for an enzootic host, the California ground squirrel (*Spermophilus beecheyi*). We sequentially remove or reduce each transmission route to understand how the influence of each route may vary between characteristic epizootic and enzootic hosts. We also use sensitivity analysis of model parameters to quantify the importance of transmission routes across a broader range of species and to explore how previously identified host characteristics interact with transmission to improve prediction of plague dynamics.

## Methods

We developed an ordinary differential equation (ODE) model consisting of both host and flea submodels (Eqs. 1–11; Figs. S1 and S2). Host and flea classes and model parameters are defined in Tables 1 and 2, respectively.

**Table 1:** Host and flea variables.

Variable	Description
*S*	Susceptible host
*E*	Exposed host
*I*	Infectious host (i.e., bacteremia ≥10^6^ cfu/mL [52], [53])
*R*	Resistant host
*M*	Infectious carcass reservoir
*N*	Population size (i.e., *S+E+I+R*)
*F_SQ_*	Susceptible, questing flea
*F_SH_*	Susceptible, on-host flea
*F_EQ_*	EP1, questing flea reservoir
*F_EH_*	EP1, on-host flea in booster-feed infection cycle
*F_LQ_*	EP2, questing flea reservoir
*F_LH_*	EP2, on-host flea in booster-feed infection cycle
*F_0_*	Breeding, on-host fleas (i.e., *F_SH_+F_EH_+F_LH_*)

**Table 2:** Parameter values.

Parameter	Epizootic host^1^	Enzootic host^2^	Description^3^	Reference
*r*	0.087	0.025	Intrinsic rate of increase	[54], [55]
*K*	200	26	Carrying capacity	[54], [55]
*μ*	0.0002	0.0005	Natural mortality rate	[54], [56]
*β_r_*	0.073	0.073	Transmission rate: infectious carcasses	[13]
*B*	20	50	Spatial correction factor to transmission	[13], [55], [56]
*σ*	0.22	0.169	(Exposed period)^−1^	[57], [58]
*α*	0.5	0.5	Disease induced mortality rate	[53], [57]
*λ*	0.091	0.091	Infectious carcass decay rate	[29]
*p*	0.01	0.412	Probability of gaining resistance	[58]
*ϕ*	0.011	0.002	Rate resistance is lost	[59]
*β_E_*	0.044	0.082	Transmission rate: EP1	[14], [18], [19]
*β_L_*	0.01	0.059	EP2 transmission rate	[18]–[20]
*δ*	0.059	0.059	Rate of leaving hosts	[60]
*a*	0.02	0.02	Questing efficiency	See Text S2
*μ_F_*	0.01	0.01	Natural mortality rate	[61]
*r_F_*	2.5	2.5	Conversion efficiency	See Text S2
*γ*	0.84	0.92	Transmission rate: hosts to vector	[14], [18], [19]
*θ_E_*	1	0.25	Rate of transition from EP1 to EP2 while feeding	[18]–[20]
*θ_L_*	1	0.33	Rate of transition from EP2 to susceptible while feeding	[18]–[20]

1 Parameterized for the black-tailed prairie dog and *Oropsylla hirsuta* system.

2 Parameterized for the California ground squirrel and *O. montana* system.

3 Units for rates are in (days)^−1^.

### Host Submodel



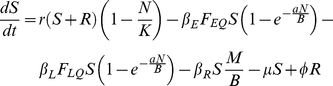
(1)

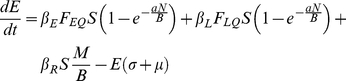
(2)

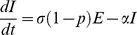
(3)

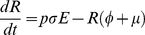
(4)

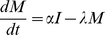
(5)


### Flea Submodel



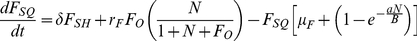
(6)

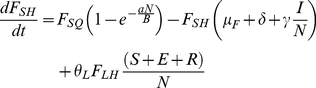
(7)

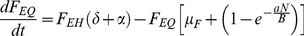
(8)

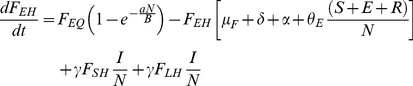
(9)

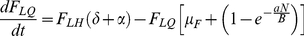
(10)

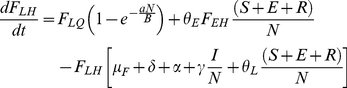
(11)


We include three transmission routes in the model (Table 3). We separate fleas into questing (i.e., host seeking) and on-host classes to differentiate between the fleas in the questing reservoir and those fleas actively participating in the booster-feed infection cycle. Transmission from both the flea reservoir and the booster-feed infection cycle occurs via early-phase transmission (EP), which is divided into two-stages. EP stage 1 (EP1) defines transmission immediately following an infectious blood meal, with transmission efficiency quickly declining as a blood meal is taken from a non-infectious host (i.e. *S*, *E*, or *R*) causing fleas to transition to EP stage 2 (EP2; [20]). Another non-infectious blood meal is required to clear infection, and consequently, infectious questing fleas remain infectious indefinitely in our model (see Table 3 and Text S1 for a test of this assumption). Also, given that social structure within a local population may be important for transmission [31], some characterization of social segregation was needed. Rather than develop a fully spatial model, we introduced a correction factor, *B*, to the transmission terms to account for heterogeneous mixing between social groups. Due to the relative ineffectiveness of blocked-flea and pneumonic transmission in a similar model [13], we ignore these routes.

**Table 3:** Transmission routes.

Mechanism	Transmission Type^1^	Influential Parameters	Testing Method
Booster-feed infection cycle	Frequency-dependent	*β_E_*, *β_L_*, *θ_E_*, *θ_L_*	Set *β_E_* = *β_L_* = 0^2^
Infectious, questing flea reservoir	Frequency-dependent	*a*, *μ_F_*, *α*, *δ* (and indirectly *β_E_* and *β_L_*)	Allow loss of infectiousness in questing fleas (see Text S1)
Infectious carcasses	Density-dependent	*β_r_*, *λ*	Set *β_R_* = 0

1 See [13].

2 Notice that the removal of booster-feed infections also requires removal of transmission from the flea reservoir.

We developed a stochastic realization of our model using C++ based on Gillespie's Direct Algorithm [11], [32]. The stochastic model was run for 300,000 events, which equates to 2–5 years. All model runs were started with a host population close to carrying capacity. Results of 100 simulations were used to obtain extinction (i.e., host population goes extinct during the model run), enzootic (i.e., both the host population and plague persist throughout the model run), and disease fade probabilities (i.e., plague goes extinct despite persistence of host population).

To understand how transmission varies between epizootic and enzootic cycles, we parameterized the model to simulate both an epizootic and enzootic population (Table 2). Parameter values for the epizootic host were based on black-tailed prairie dogs and *Oropsylla hirsuta*, a common prairie dog flea [18], [21], [33]. The enzootic host parameter values reflected California ground squirrels and their dominant flea species, *O. montana* [1], [34], [35]. We also parameterized the flea sub-model for *O. tuberculata cynomuris*, another common prairie dog flea (Table S1, [18]), but the results for this species were similar to those for *O. hirsuta*. All parameter values were obtained from the literature or fit to observed data, but when data were not available, we substituted for the most closely related species available (for details of parameter estimation see Text S2).

To test the importance of transmission routes, we systematically removed them from both the epizootic and enzootic systems (Table 3). Because all flea-borne transmission is tied to early-phase transmission efficiency in our model, booster feeds cannot be removed without removing the flea reservoir. By comparing behavior with no flea-borne transmission to behavior when only infectious questing fleas were removed, we were able to determine the effect of the booster-feed infection cycle on plague dynamics. The simulations with flea-borne transmission began with five questing EP1 and EP2 fleas, and simulations with no flea-borne transmission began with one infectious carcass.

We also examined the relative importance of the transmission routes in a more general sense by performing sensitivity analysis on model parameters associated with each transmission route (Table 3). By extending this sensitivity analysis to include model parameters that represent characteristics of the hosts and fleas, we determined how previously identified host characteristics may interact with transmission routes to determine plague dynamics. We used a multi-parameter sensitivity analysis proposed by Blower and Dowlatabadi [36]. We constructed 100 random parameter sets using stratified random samples from uniform distributions spanning a range of potential values for host and flea species. The range was determined by increasing the largest value of a parameter found in our parameter sets (Tables 1 and S1) by an order of magnitude, which is a reasonable approximation of the range for most parameter values. Each parameter set was simulated 100 times in the full model. Partial-rank correlation coefficients (PRCCs) between each parameter and model output determined the relative importance of each parameter.

## Results

**Figure 1:**
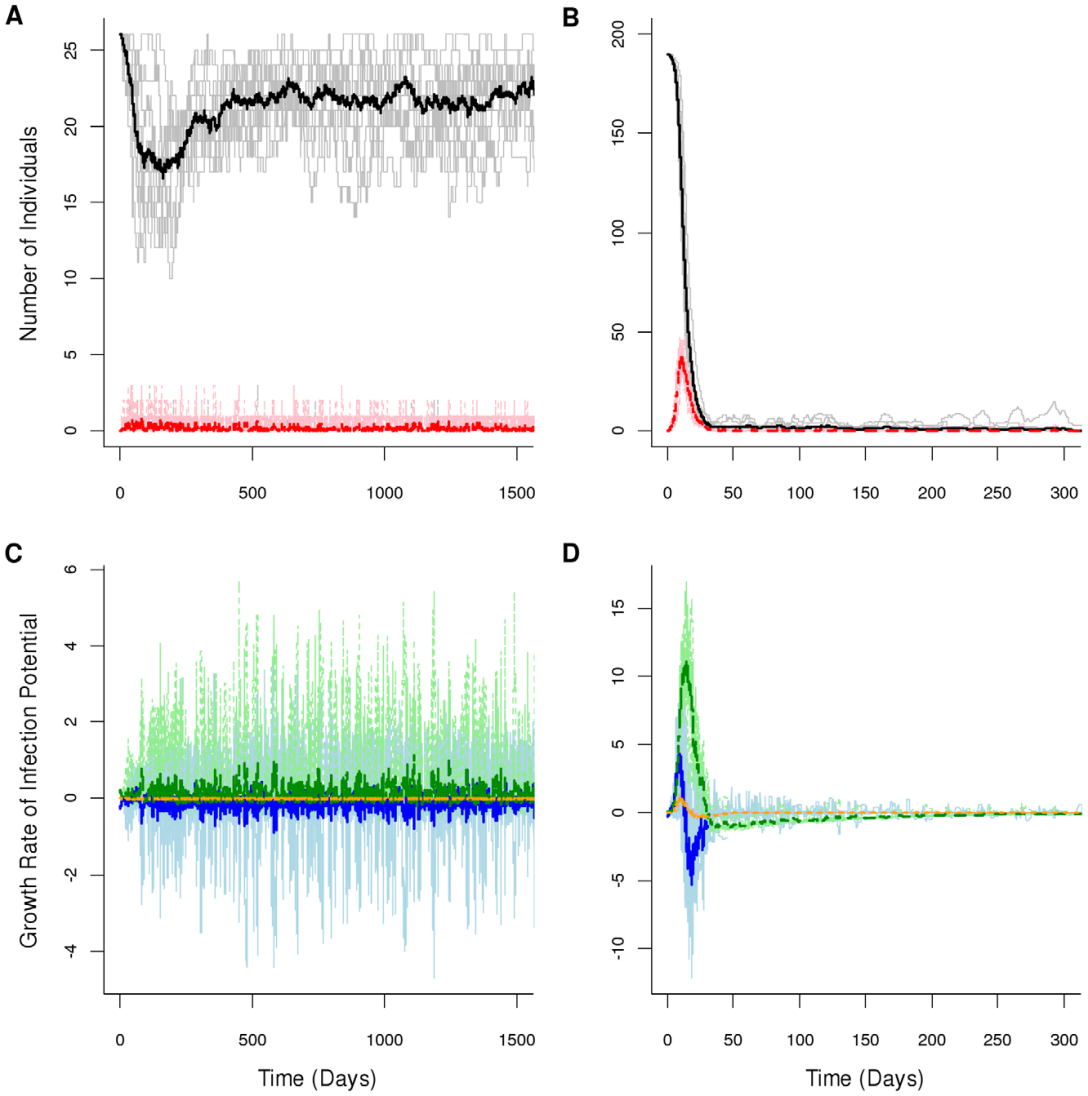
Enzootic and epizootic plague dynamics. Model behavior for given parameter values (Table 2) with light/bold lines giving results for independent model runs/average behavior. Total population size (gray/black) and number of infectious individuals (pink/red) are shown for the A) enzootic host and B) epizootic host. The growth rate of infection potential over time for the three transmission routes, booster-feed infections (light blue/blue), questing flea reservoir (light green/green), and carcass reservoir (light orange/orange) are shown for the C) enzootic host and D) epizootic host.

The model showed clear enzootic and epizootic behavior for our two parameterizations (Fig. 1 A and B respectively). In addition, model results for the prairie dog and ground squirrel parameterizations closely matched independent data (for detailed model results see Text S3). Parameter values for California ground squirrels created enzootic behavior for prolonged periods (>3 years; Fig. 1A) with 90% of surviving hosts found to be resistant to plague. Similarly, a natural population of California ground squirrels showed evidence of antibody responses to previous plague exposure in 11 of 13 years, accounting for 93% of the total population [35]. For the prairie dog parameter set, the model predicted high extinction probabilities similar to areas where epizootics have been observed on black-tailed prairie dog towns [37], as well as others specifically studied by us on the Pawnee National Grassland where all 12 confirmed plague epizootics on towns from 2003 to 2008 resulted in severe population declines or extinction (Fig. 1B). The model also predicted short-lived epizootics with towns declining to near extinction after about 3 weeks with remnant hosts persisting for around 37.5 weeks (Fig. 1B), a range inclusive of the observed 6–8 week window from first detection of plague to apparent town extinction [13].

Looking at the role of our three transmission routes during enzootic cycles, infection potential from the booster-feed infection cycle declines during the majority of the model run (i.e., negative growth rate of infection potential), while the infectious, questing flea reservoir increases almost throughout (Fig. 1C). Infectious carcasses played little role in the enzootic cycle (Fig. 1C). In contrast, infection potential from booster-feeds showed a sharp increase during the early-stages of an epizootic, but then quickly declined as the epizootic progressed (Fig. 1D). Infection potential from the flea reservoir showed a similar pattern, although it continued to increase after infection from the booster-feed infection cycle crashed (Fig. 1D). The role of infectious carcasses paralleled that of the flea reservoir although the magnitude of change was not as great (Fig. 1D).

**Figure 2:**
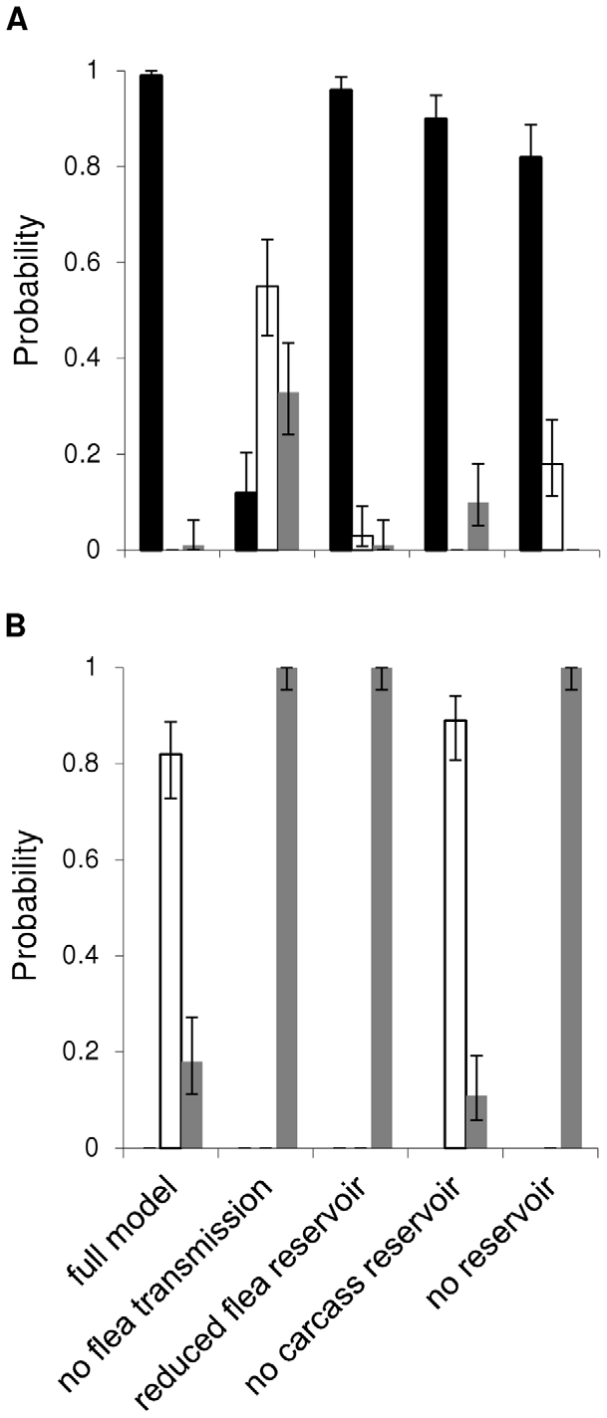
Transmission hypothesis testing. Testing was done using default parameter values for the (a) black-tailed prairie dog and (b) California ground squirrel. Each case represents the effect of removing either one or multiple transmission routes on extinction probability (black bars), enzootic probability (white bars), or disease fade probability (gray bars). 95% confidence intervals are also given.

Systematic removal of transmission routes helped provide a clearer picture of each in plague dynamics, especially for epizootic behavior (Fig. 2). For the epizootic host parameterization, removing all flea-borne transmission (i.e., booster-feeds and the flea reservoir) resulted in a shift to enzootic behavior (Fig. 2A). However, when only the infectious, questing flea reservoir is removed, model behavior is again dominated by epizootics (Fig. 2A). Combined, these results suggest that booster-feed transmission plays an important role in epizootics. Removal of the carcass reservoir still results in primarily epizootics, but disease fade is more likely (Fig. 2A). Removal of both reservoirs shows a significant increase in enzootic behavior with epizootics still dominating (Fig. 2A). Overall, this supports the idea that booster-feed transmission dominates but at least one type of reservoir transmission is needed to reach epizootic levels. In the enzootic host, removal of all flea-borne transmission resulted in a shift to disease fade-out (Fig. 2B). However, when booster-feeds were reinserted into the model and only the flea reservoir was removed, the shift to disease fade-out remained (Fig. 2B). Removal of the carcass reservoir alone has little impact on plague dynamics (Fig. 2B). Together, these results on enzootic probability suggest a consistent role for a flea reservoir in enzootic dynamics.

**Figure 3:**
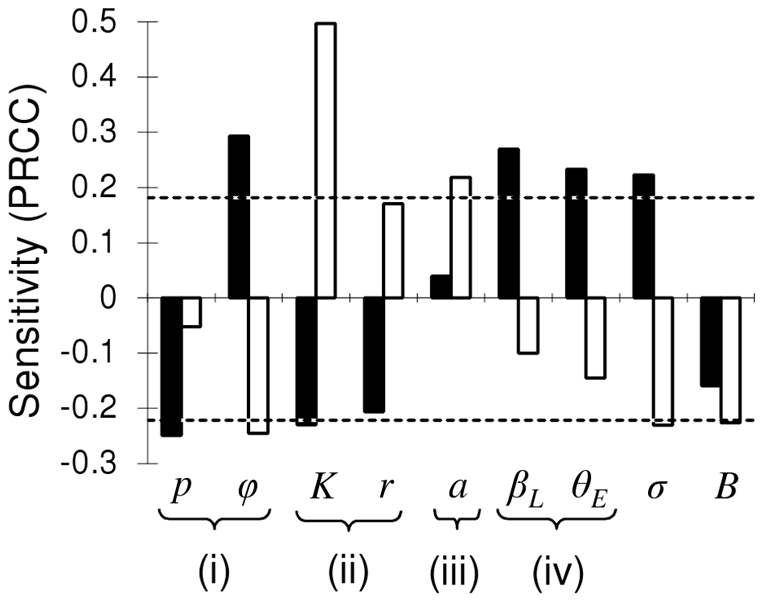
Multi-parameter sensitivity analysis. Partial rank correlation coefficients (PRCCs) between extinction (black bars) and enzootic (white bars) probabilities and model parameters. Dashed lines indicate the critical values for significance (p<0.05). Parameters are grouped by the following: (i) host resistance, (ii) host population size, (iii) efficiency of the flea reservoir, and (iv) efficiency of the booster-feed infection cycle.

Our multi-parameter sensitivity analysis was consistent with the relative importance of transmission routes described above and revealed that model results were sensitive to parameters influential to both the booster-feed infection cycle and the infectious, questing flea reservoir (Table 3; Fig. 3). In particular, flea questing efficiency, *a*, was positively correlated with enzootic probability but had little effect on extinction probability. Increasing transmission efficiency from EP2, *β_L_*, increased extinction probability as did an increase in the transition rate between EP1 and EP2 for fleas taking non-infectious blood meals, *θ_E_*.

Population responses to plague infection were also sensitive to several host parameters in the model (Fig. 3). Among these, extinction probability was increased by higher rates of resistance loss, *ϕ*, and shorter host exposure periods (i.e., increased values of *σ*). However, increased host resistance, *p*, and increased host carry capacity, K, served to decrease epizootic behavior. In contrast, enzootic probability was increased by increasing host carrying capacity and declined with higher rates of resistance loss, shorter host exposure periods, and decreasing host connectance (i.e., increased values of our of spatial correction factor, *B*). Sensitivities that are not reported were not significant.

## Discussion

Our model produced characteristic enzootic and epizootic behaviors, and model behaviors for our specific parameterizations were consistent with empirical observations of plague activity in the hosts they were based on, black-tailed prairie dogs and California ground squirrels. The agreement with natural systems highlights our ability to reliably compare the shifting roles of transmission routes in creating each dynamic. In particular, the booster-feed infection cycle is primarily responsible for epizootic behavior. While laboratory experiments have demonstrated that the booster feed infection cycle results in the maintenance of infection levels in fleas [20], we extend this result and show here that the booster-feed infection cycle can produce sustained transmission capable of initiating large scale epizootics (Figs. 1D and 2A). However, booster-feed infections may rapidly reduce the host population making prolonged periods in the booster-feed infection cycle unlikely due to host limitation. Consequently, an additional source of infection (i.e., infectious carcasses or infectious, questing fleas) is most likely needed to ensure extinction of remnant populations in epizootic hosts (Figs. 1D and 2A). In contrast, enzootic dynamics rely on a shift from the continuous maintenance of transmission chains through the booster-feed infection cycle seen in epizootic dynamics to the buildup of infectious, questing fleas (Figs. 1C and 2B).

Our sensitivity analysis supports the role of shifting transmission dynamics in determining plague dynamics in the host population. We found that epizootic behavior (i.e., higher extinction probability) was strongly affected by flea characteristics that determine both the strength and turnover rate of the booster-feed infection cycle, while enzootic potential was strongly influenced by flea questing efficiency adding support to the involvement of a flea reservoir in the maintenance of plague at the population level [38]. While the strength of transmission routes varies between epizootics and enzootics, it is important to note that our sensitivity analysis suggests that transmission in general is tempered by heterogeneous mixing of individuals (i.e., higher values of our spatial correction factor, *B*; Fig. 3). Understanding variation in the strength of transmission routes is thus highly contingent upon understanding the processes that determine epizootiologically relevant mixing of hosts. Recent modeling studies have revealed the potential for alternate hosts, like grasshopper mice, to serve as a link between spatially distinct prairie dog coteries [31]. Additionally, occasional non-local interactions between socially distinct groups of individuals could increase their epizootiological connection leading to more global connectivity as seen in a population of African lions [39]. The importance of a flea reservoir in plague dynamics also supports the idea that a questing flea reservoir could increase connectance between individuals by linking socially distinct units through transient interactions with a common infectious reservoir. However more research is needed to determine mechanisms governing connectivity and their role in determining the relative importance of transmission routes.

While the flea reservoir may be important in connecting spatially distinct groups of hosts, we also hypothesize that questing fleas may act as a bridge in enzootics, connecting temporally separated pools of susceptible hosts generated from a resistant refuge. This endogenously derived temporal bridge contrasts with more traditionally hypothesized exogenous sources of re-infection. Bat rabies virus may display a similar endogenous bridging mechanism by entering a quiescent state during host hibernation, thus creating a bridge between birth pulses that refresh the susceptible pool [40], [41]. Additionally, other systems are consistent with an endogenous temporal bridging mechanism including leptospirosis epizootics in California sea lions [42], overwintering dynamics in other vector-borne diseases like bluetongue virus in northern Europe [43] and West Nile Virus in the eastern United States [44], and transstadial transmission of Lyme disease spirochetes [45].

Most of the previous research on the variability in population responses to plague infection has focused on host traits, and our sensitivity analysis confirmed some of these observations, particularly the importance of host resistance and population size as observed in Asian great gerbils [4], [6]–[8]. This result is not surprising given the extensive evidence for a critical community size in the theoretical disease literature (e.g., [9], [10], [46], [47]) and seen in other systems, such as measles [48], [49], phocine distemper [50], and cowpox virus [51].

However, while our analysis confirms previous observations on the role of host characteristics in determining disease dynamics, it is important to note that these traits do not act independently of transmission routes to determine population response and thus, the effects of host traits may depend on the specific transmission routes operating. For example, we found that increasing host carrying capacity generally increased enzootic potential in our sensitivity analysis. However, our specific results for prairie dogs and California ground squirrels exhibited the opposite of the expected responses with black-tailed prairie dogs having larger population sizes but higher probabilities of extinction. Here, knowledge of transmission shifts may be more informative. Specifically, the importance of booster-feeds in epizootics, a transmission route that relies on continued contact between hosts and fleas, may create a situation where increasing host abundance leads to large epizootic potential that cannot be maintained. This is in contrast to enzootic hosts where an endogenous bridging mechanism like infectious, questing fleas overcomes issues of host limitation. The maintenance of infection potential in a flea reservoir may also alter the traditionally hypothesized role of resistance in promoting enzootics. In this case, resistance may primarily be important in avoiding epizootics and becomes important in promoting enzootics only when infectious, questing fleas dominate transmission. Thus, host and flea characteristics may scale up through transmission routes allowing for more robust predictions than when considering either host or flea characteristics alone.

## Supporting Information

Figure S1 **Flow chart for the host sub model.** The three transmission routes included in the model are highlighted: booster-feed infection cycle (blue), infectious, questing flea reservoir (green), and infectious carcasses (orange).
(TIF)

Figure S2 **Flow chart for the flea submodel.** The relationship between the booster-feed infection cycle (blue) and infectious flea reservoir (green) is highlighted.
(TIF)

Table S1 **Alternate flea parameter values.** Parameter values for the prairie dog flea *O. tuberculata cynomuris*. Other flea species are provided for comparison.
(DOC)

Text S1 **Alternate flea submodel which prevents the buildup of infectious, questing fleas.**
(DOC)

Text S2 **Parameter fitting and estimation.**
(DOC)

Text S3 **Detailed prairie dog and California ground squirrel model outputs.**
(DOC)
