# Supplementary figures and images for: Transmission Shifts Underlie Variability in Population Responses to Yersinia pestis Infection
Source: PLoS One. 2011 Jul 25;6(7):e22498. doi: 10.1371/journal.pone.0022498 (PMC3143141; doi:10.1371/journal.pone.0022498)

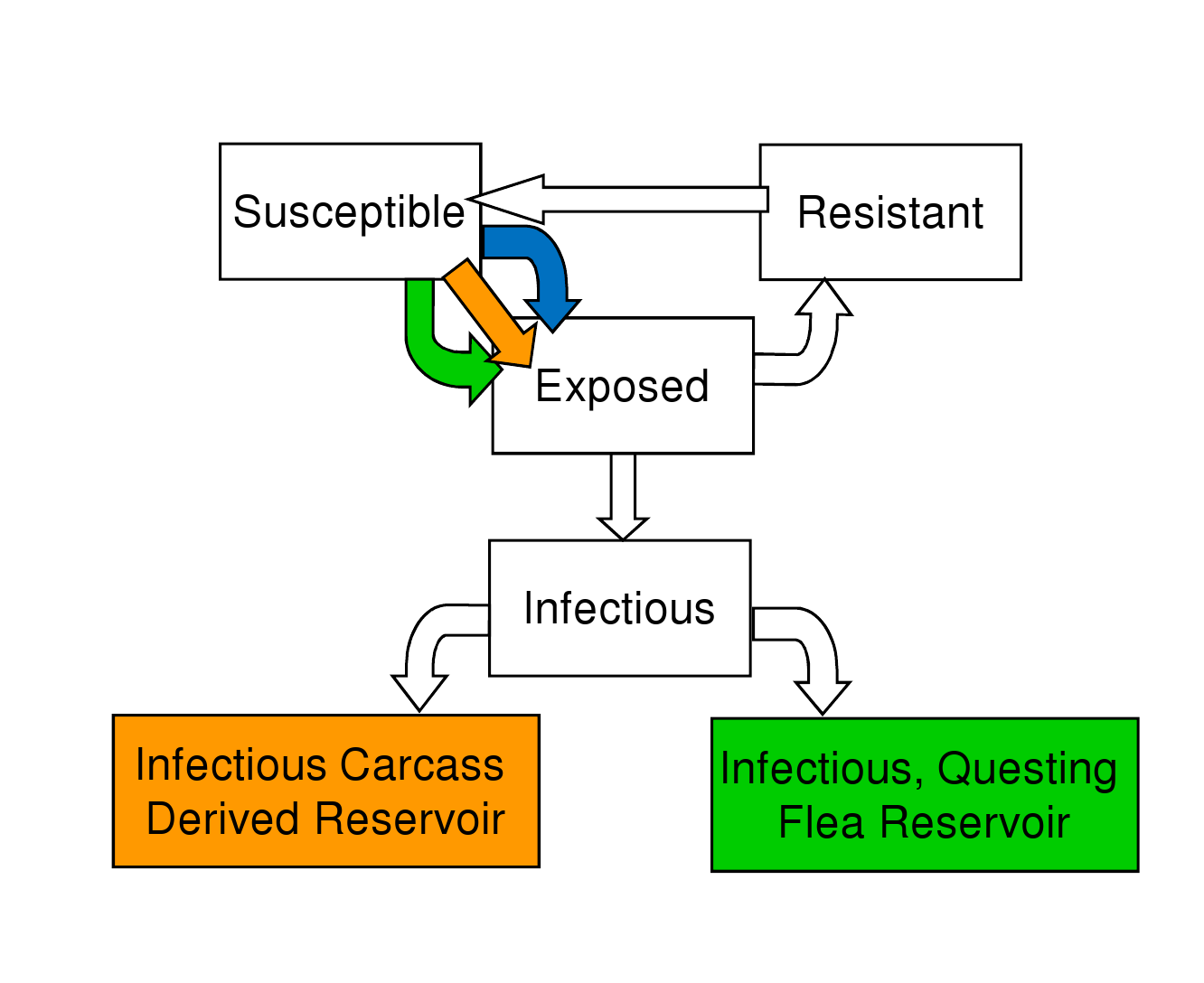

Supplement: Figure S1 — Flow chart for the host sub model. The three transmission routes included in the model are highlighted: booster-feed infection cycle (blue), infectious, questing flea reservoir (green), and infectious carcasses (orange). (TIF) [file pone.0022498.s001.tif]

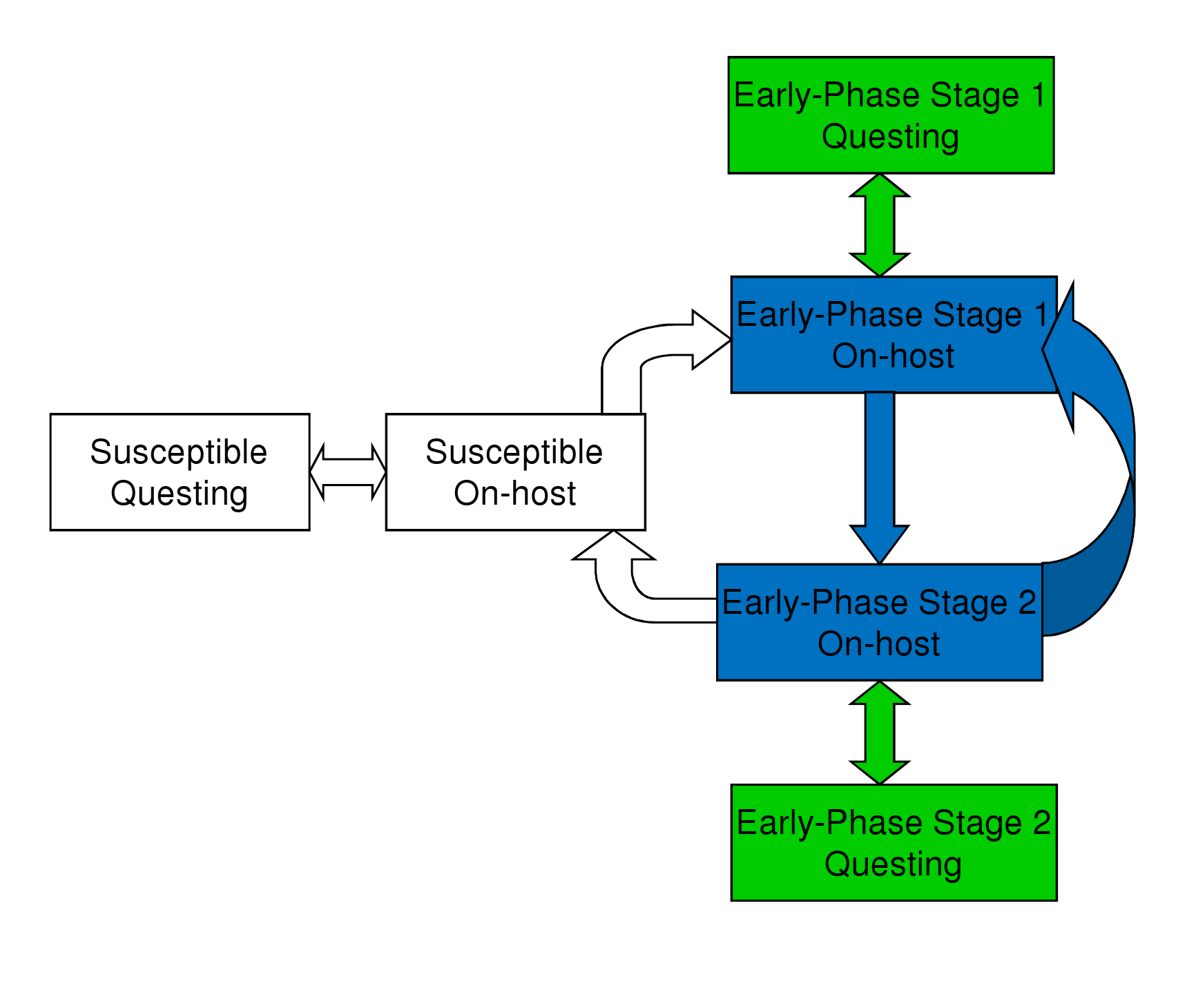

Supplement: Figure S2 — Flow chart for the flea submodel. The relationship between the booster-feed infection cycle (blue) and infectious flea reservoir (green) is highlighted. (TIF) [file pone.0022498.s002.tif]
