# Supplementary material for: Transmission Shifts Underlie Variability in Population Responses to Yersinia pestis Infection
Source: PLoS One. 2011 Jul 25;6(7):e22498. doi: 10.1371/journal.pone.0022498 (PMC3143141; doi:10.1371/journal.pone.0022498)
Supplement: Table S1 — Alternate flea parameter values. Parameter values for the prairie dog flea O. tuberculata cynomuris. Other flea species are provided for comparison. (DOC) [file pone.0022498.s003.doc]

**Table S1: Alternate flea parameter values**. Parameter values for the prairie dog flea *O. tuberculata cynomuris*. Other flea species are provided for comparison.

| Parameter | *Oropsylla hirsuta* | *O. tuberculata cynomuris* | *O. montana* | Description^1^ | Reference |
| --- | --- | --- | --- | --- | --- |
| *β_E_* | 0.044 | 0.147 | 0.082 | Transmission rate: EP1 | [1-3] |
| *β_L_* | 0.01 | 0.01 | 0.059 | EP2 transmission rate | [1,2,4] |
| *δ* | 0.059 | 0.059 | 0.059 | Rate of leaving hosts | [5] |
| *a* | 0.02 | 0.02 | 0.02 | Questing efficiency | See Text S2 |
| *μ_F_* | 0.01 | 0.01 | 0.01 | Natural mortality rate | [6] |
| *r_F_* | 2.5 | 2.5 | 2.5 | Conversion efficiency | See Text S2 |
| *γ* | 0.84 | 0.82 | 0.92 | Transmission rate: hosts to vector | [1-3] |
| *θ_E_* | 1 | 1 | 0.25 | Rate of transition from EP1 to EP2 while feeding | [1,2,4] |
| *θ_L_* | 1 | 1 | 0.33 | Rate of transition from EP2 to susceptible while feeding | [1,2,4] |

^1^Units for rates are in days.

**References**

1. Wilder AP, Eisen RJ, Bearden SW, Montenieri JA, Tripp DW, et al. (2008) Transmission efficiency of two flea species (*Oropsylla tuberculata cynomuris* and *Oropsylla hirsuta*) involved in plague epizootics among prairie dogs. EcoHealth 5: 205-212.

2. Wilder AP, Eisen RJ, Bearden SW, Montenieri JA, Gage KL, et al. (2008) *Oropsylla hirsuta* (Siphonaptera: Ceratophyllidae) can support plague epizootics in black-tailed prairie dogs (*Cynomuris ludovicianus*) by early-phase transmission of *Yersinia pestis*. Vector-Borne Zoonot 8: 359-368.

3. Eisen RJ, Bearden SW, Wilder AP, Montenieri JA, Antolin MF, et al. (2006) Early-phase transmission of *Yersinia pestis* by unblocked fleas as a mechanism explaining rapidly spreading plague epizootics. Proc Natl Acad Sci U S A 103: 15380-15385.

4. Eisen RJ, Lowell JL, Montenieri JA, Bearden SW, Gage KL (2007) Temporal dynamics of early-phase transmission of *Yersinia pestis* by unblocked fleas: secondary infectious feeds prolong efficient transmission by *Oropsylla montana* (Siphonaptera: Ceratophyllidae). J Med Entomol 44: 672-677.

5. Hartwell WV, Quan SF, Scott KG, Kartman L (1958) Observations on flea transfer between hosts: a mechanism in the spread of bubonic plague. Science 127: 814.

6. Eskey CR, Haas VH (1940) Plague in the western part of the United States. Publ

Health Bull 254: 1-82.
