# Supplementary material for: Transmission Shifts Underlie Variability in Population Responses to Yersinia pestis Infection
Source: PLoS One. 2011 Jul 25;6(7):e22498. doi: 10.1371/journal.pone.0022498 (PMC3143141; doi:10.1371/journal.pone.0022498)
Supplement: Text S1 — Alternate flea submodel which prevents the buildup of infectious, questing fleas. (DOC) [file pone.0022498.s004.doc]

**Text S1: Alternate flea submodel which prevents the buildup of infectious, questing fleas.**

In our original flea submodel, we assumed fleas must take a non-infectious blood meal to clear infection. Thus upon leaving a host, fleas maintained their current infection status indefinitely. We also developed an alternate version of the flea submodel that allowed fleas to clear infection without a feeding requirement (Eqs. S1-S6). This version of the flea submodel enabled the infectious questing flea reservoir to wane over time and allowed us to test the role of the questing flea reservoir on plague dynamics.

We defined the following additional parameters and their default values as: *ε* = rate at which fleas in EP1 transition to EP2 when not feeding = 0.125 [1]; *ξ* = rate at which fleas in EP2 clear infection when not feeding = 0.033 [2]. Default values were determined according to infectious periods seen in studies that maintained infectious flea stocks through continued blood meals. As such, these parameter values represent rates that may be much faster than those that would be observed in natural questing fleas that may enter a quiescent state when not feeding. All other parameters were given their default values (Tables 2 and S1).

*Alternate Flea Submodel*

$\frac{dF_{SQ}}{dt}=\delta F_{SH}+r_{F}F_{O}\left(\frac{N}{1+N+F_{O}}\right)-F_{SQ}\left[\mu_{F}+\left(1-e^{-\frac{aN}{B}}\right)\right]+\xi F_{LQ}$ (S1)

$\frac{dF_{SH}}{dt}=F_{SQ}\left(1-e^{-\frac{aN}{B}}\right)-F_{SH}\left(\mu_{F}+\delta+\gamma\frac{I}{N}\right)+\theta_{L}F_{LH}\frac{\left(S+E+R\right)}{N}$ (S2)

$\frac{dF_{EQ}}{dt}=F_{EH}\left(\delta+\alpha\right)-F_{EQ}\left[\mu_{F}+\left(1-e^{-\frac{aN}{B}}\right)\right]-\varepsilon F_{EQ}$ (S3)

$\frac{dF_{EH}}{dt}=F_{EQ}\left(1-e^{-\frac{aN}{B}}\right)-F_{EH}\left[\mu_{F}+\delta+\alpha+\theta_{E}\frac{\left(S+E+R\right)}{N}\right]+\gamma F_{SH}\frac{I}{N}+\gamma F_{LH}\frac{I}{N}$ (S4)

$\frac{dF_{LQ}}{dt}=F_{LH}\left(\delta+\alpha\right)-F_{LQ}\left[\mu_{F}+\left(1-e^{-\frac{aN}{B}}\right)\right]+\varepsilon F_{EQ}-\xi F_{LQ}$ (S5)

$\frac{dF_{LH}}{dt}=F_{LQ}\left(1-e^{-\frac{aN}{B}}\right)+\theta_{E}F_{EH}\frac{\left(S+E+R\right)}{N}-F_{LH}\left[\mu_{F}+\delta+\alpha+\gamma\frac{I}{N}+\theta{}_{L}\frac{\left(S+E+R\right)}{N}\right]$ (S6)

**References**

1. Eisen RJ, Lowell JL, Montenieri JA, Bearden SW, Gage KL (2007) Temporal dynamics of early-phase transmission of *Yersinia pestis* by unblocked fleas: secondary infectious feeds prolong efficient transmission by *Oropsylla montana* (Siphonaptera: Ceratophyllidae). J Med Entomol 44: 672-677.

2. Engelthaler DM, Hinnebusch BJ, Rittner CM, Gage KL (2000) Quantitative competitive PCR as a technique for exploring flea-*Yersinia pestis* dynamics. Am J Trop Med Hyg 62: 552-560.
