# Supplementary material for: Transmission Shifts Underlie Variability in Population Responses to Yersinia pestis Infection
Source: PLoS One. 2011 Jul 25;6(7):e22498. doi: 10.1371/journal.pone.0022498 (PMC3143141; doi:10.1371/journal.pone.0022498)
Supplement: Text S2 — Parameter fitting and estimation. (DOC) [file pone.0022498.s005.doc]

Text S2: Parameter fitting and estimation.

Flea questing efficiency, *a*, and conversion efficiency, *r_F_*, were fit to observed flea loads on California ground squirrels (observed = 28.4 [1]; model = 30.4). Because *a* and *r_F_* had little impact on a previous prairie dog model [2] and are not well characterized in general [3], we assumed that these parameters were similar for all flea species but also tested their importance using sensitivity analysis. The transmission correction factor, B, was estimated from the home range sizes of hosts [2,4].

**References**

1. Eskey CR, Haas VH (1940) Plague in the western part of the United States. Publ

Health Bull 254: 1-82.

2. Webb CT, Brooks CP, Gage KL, Antolin MF (2006) Classic flea-borne transmission does not drive plague epizootics in prairie dogs. Proc Natl Acad Sci U S A 103: 6236-6241.

3. Keeling MJ, Gilligan CA (2000) Metapopulation dynamics of bubonic plague. Nature 407: 903-906.

4. Linsdale JM (1946) The California Ground Squirrel. Berkeley: University of California Press. 475 p.
