# Supplementary material for: Transmission Shifts Underlie Variability in Population Responses to Yersinia pestis Infection
Source: PLoS One. 2011 Jul 25;6(7):e22498. doi: 10.1371/journal.pone.0022498 (PMC3143141; doi:10.1371/journal.pone.0022498)
Supplement: Text S3 — Detailed prairie dog and California ground squirrel model outputs. (DOC) [file pone.0022498.s006.doc]

**Text S3: Detailed prairie dog and California ground squirrel model outputs.**

For the prairie dog parameters, the model predicted an extinction probability of 0.99 (95% CI [0.97, 1]) with an average time to extinction of 263 days (95% CI [204, 322]) and an epizootic time (i.e., time to reduce the host population to 1/10 of its carrying capacity) of 21.48 days (95% CI [21.13, 21.83]). No prairie dog colonies reached an enzootic state, and plague faded out in one of the 100 runs after only 6 days.

In the ground squirrel system, no extinctions were observed. The probability of enzootic persistence was 0.82 (95% CI [0.74, 0.90]) with 90.3% of individuals resistant to infection (95% CI [88.8, 91.8]). The probability of disease fade-out was 0.18 (95% CI [0.10, 0.26]) with an average time to fade-out of 538 days (95% CI [415, 661]).
